# Squamous cell carcinoma and basal cell carcinoma of the lips: 25 years of experience in a northeast Brazilian population

**DOI:** 10.4317/medoral.26454

**Published:** 2024-05-25

**Authors:** Weslay Rodrigues da Silva, Manoela Moura de Bortoli, Sérgio Ricardo Silveira Leite, Caio César da Silva Barros, Maria de Fátima Medeiros Brito, Luciano Tavares Montenegro, Ricardo José Holanda Vasconcellos, Deborah Pitta Paraíso Iglesias, Ana Paula Veras Sobral

**Affiliations:** 1 School of Dentistry, Postgraduate program in Dentistry, University of Pernambuco (UPE), Recife, PE, Brazil; 2 Oswaldo Cruz University Hospital (UPE), Integrated Anatomic Pathology Center, Recife, PE, Brazil; 3 DDS, Research collaborator, School of Dentistry, Federal University of Rio Grande do Norte (UFRN), Natal, RN, Brazil; 4 DDS MSc, PhD, Federal University of Rio Grande do Norte (UFRN), Natal, RN, Brazil; 5 Departament of Tropical Medicine, Federal University of Pernambuco (UFPE), Recife, PE, Brazil; 6 Department of Pathology, Federal University of Pernambuco (UFPE), Recife, PE, Brazil; 7 Postgraduate Program in Oral and Maxillofacial Surgery, School of Dentistry, University of Pernambuco (UPE), Recife, PE, Brazil

## Abstract

**Background:**

The lips are the transition zone between the facial skin and the oral mucosa and are the site of alterations related to a broad spectrum of etiologies. Squamous cell carcinoma (SCC) and basal cell carcinoma (BCC) are the most prevalent neoplasms affecting lips. This study evaluated the demographic and clinicopathological features of the SCC and BCC in the lip.

**Material and Methods:**

A retrospective cross-sectional descriptive study (1994-2019) was carried out. Demographic and clinicopathologic data were collected from a hospital’s dermatological service and an oncologic hospital. The data were submitted to descriptive analysis and Pearson's chi-square and Fisher's exact tests (*p* ≤ 0.05).

**Results:**

417 medical records were analyzed, of which 323 corresponded to SCC (77.5%) and 94 to BCC (22.5%). SCC showed more frequency in males (58.8%) and BCC in females (54.3%). The lower lip was significantly affected in male patients (*p* < 0.0001) and by both neoplasms (70.6% and 56.4%, respectively; *p* = 0.014). SCC and BCC were mainly treated with surgery (88.3% and 93.2%, respectively). Surgical margin was frequently negative in SCC and BCC (87%; 72.3%, respectively), and no recurrence was observed in 79.9% of SCC and 69.1% of BCC cases.

**Conclusions:**

SCC was more frequent in male patients, while BCC showed more frequency in female patients. Both neoplasms mainly affect the lower lip. Understanding the epidemiological profile of these lesions in the lip, as well as their etiology and clinical features, is fundamental for appropriate clinical conduct and the creation and/or amplification of preventive measures.

** Key words:**Epidemiology, oral pathology, oral mucosal lesions.

## Introduction

The lips are the anterior limit of the oral cavity and consist of the transition zone between the facial skin and the oral mucosa (1). These anatomical structures play an important role in facial expression, chewing, phonation, and tactile sensation and contribute to facial aesthetics. Also, the lips may be the site of clinical and pathological alterations related to a broad spectrum of etiologies, ranging from traumatic, inflammatory, and infectious lesions to malignant neoplasms (1-3).

Lip cancer corresponds to 23.6%-30% of all oral cavity malignant neoplasms, and the lower lip is the most frequently affected (90%), followed by the upper lip (7%) and the labial commissure (3%). Among the malignant neoplasms affecting the lip, squamous cell carcinoma (SCC) and basal cell carcinoma (BCC) are the most prevalent (4). Ultraviolet (UV) radiation is the main etiological factor for developing both the SCC and BCC in the lip. Also, these neoplasms may exhibit similar clinical presentations as hard, firm, crusted, and painless ulcers (4-6). The SCC has a favorable prognosis, with an 82.1% 5-year survival rate. However, it has a worse prognosis than BCC, whose 5-year survival rate is over 90% (7,8). In this context, we investigated the occurrence and the demographic and clinicopathological characteristics of SCC and BCC of the lip over 25 years in a dermatological service and an oncologic hospital in northeastern Brazil.

## Material and Methods

This retrospective cross-sectional study investigated the occurrence of squamous cell and basal cell carcinoma of the lips diagnosed at the Dermatology Service of Hospital das Clínicas of the Federal University of Pernambuco (UFPE) and Cancer Hospital of Pernambuco from 1994 to 2019. Demographic and clinicopathologic data regarding sex, age, anatomic site, clinical aspect, TNM grading system, histopathological diagnosis, surgical margin, and recurrence were collected from the patient’s medical records. Nevertheless, this data was not available in all SCC and BCC cases. In this context, cases needed to contain information about the histopathological diagnosis to be included in the present study. On the other hand, cases that did not affect the lips and/or had an undetermined diagnosis were excluded from the sample. Clinical cancer staging was classified according to the American Joint Committee on Cancer - Cancer Staging Manual (9).

The data were submitted for a descriptive analysis using SPSS (Statistical Package for Social Sciences; version 22.0; IBM, Chicago, IL, USA). Pearson's chi-square test was performed to assess the association between the anatomical location of the lesions and clinicopathologic characteristics (sex, histopathological diagnosis, clinical stage, and treatment). The significance level was set at 5% (*p* ≤ 0.05) for all tests.

## Results

**Figure 1:**
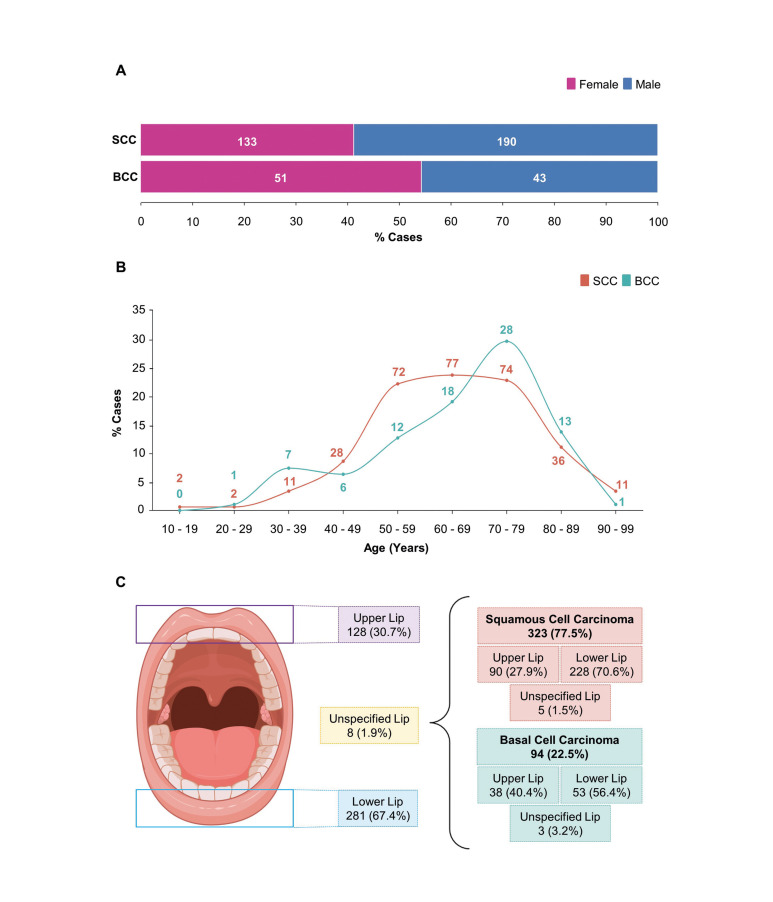
Demographic profile of squamous cell carcinoma (SCC) and basal cell carcinoma (BCC). (A) Distribution of sex showing high frequency of male patients diagnosed with SCC and female patients diagnosed with BCC (The number of cases (n) is represented inside (A) of the bars). (B) Age distribution according to the neoplasm. A high frequency of SCC was diagnosed between 50 and 79 years old, while BCC showed a high frequency between 70 and 79 years old (The number of cases (n) is represented above the dots in the lines). (C) Frequency of neoplasms diagnosed according to the anatomic site. It is observed that, together and individually, SCC and BCC affected the lower lip more.

A total of 417 patients were diagnosed with SCC and BCC during the period studied. Of these, 323 (77.5%) lip carcinomas were histopathologically diagnosed as SCC and 94 (22.5%) as BCC in both centers. It was found that SCC was diagnosed more frequently in males (58.8%; *n* = 190), while BCC was more diagnosed in females (54.3%; *n* = 51) (Fig. 1). The mean age of SCC was 64.7 ± 14.8 years old, while BCC presented a mean age of 65 ± 15.3 years old. SCC was more frequent in patients between the sixth and eighth decades of life, and BCC was more frequent in the eighth decade of life (Fig. 1).

SCC and BCC significantly affected the lower lip (70.6%; *n* = 228; 56.4%; *n* = 53, respectively; *p* = 0.014) (Fig. 1; Table 1). It was observed that BCC cases initially affected the skin, and the lesion extended to lip vermilion. Also, when analyzing the association between sex and anatomical location, it was observed that the lower lip of male patients was more affected than the upper one (*p* < 0.0001) (Table 1). Of the 417 patients, 48% reported outdoor occupational activities related to chronic sun exposure, and SCC was frequently clinically described as an ulcer and plaque. In contrast, BCC was most described as a nodule and papule.

**Figure 2:**
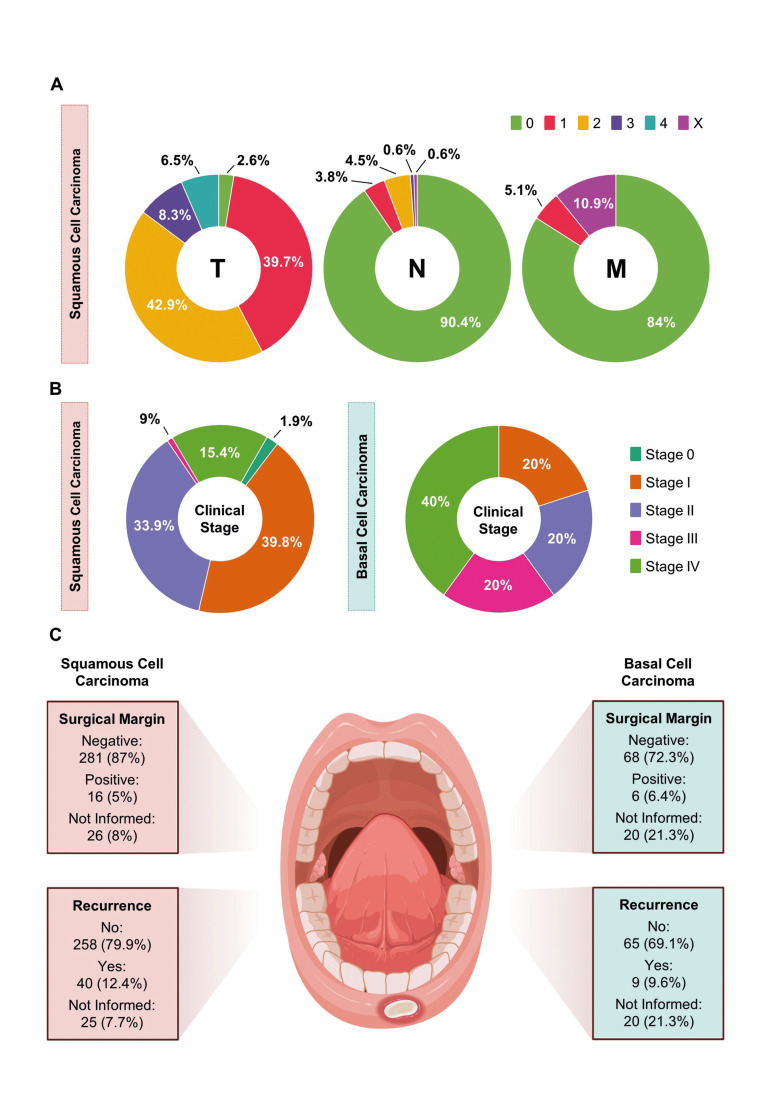
Clinicopathological characteristics of squamous cell carcinoma (SCC) and basal cell carcinoma (BCC). (A) TNM grading system information. SCC exhibited a high frequency of tumors with sizes ≤ 20 mm (T1) and > 20 mm to ≤ 40 mm (T2), as well as the absence of regional lymph node metastasis (N0) and distant metastasis (M0). The data corresponds to the medical records that presented information about the variable (SCC = 156 cases) (B) Clinical stage information. SCC exhibited a high frequency of tumors in Stages I and II, while Stage IV was the most frequent in BCC. The percentage corresponds to the medical records that presented information about the variable (SCC = 156 cases; BCC = 05 cases). (C) Surgical margin and recurrence in SCC and BCC. Both SCC and BCC exhibited a high frequency of negative surgical margins as well as the absence of recurrence of the tumor.

Concerning the TNM grading system findings, SCC cases presented a high frequency of tumors with sizes ≤ 20 mm (T1) (39.7%) and > 20 mm to ≤ 40 mm (T2) (42.9%), as well as the absence of regional lymph node metastasis (N0) (90.4%) and distant metastasis (M0) (84%) (Fig. 2). In contrast, only 05 cases of BCC presented this information. From these 05 cases, two cases presented tumors with size > 20 mm to ≤ 40 mm (T2), one case with size ≤ 20 mm (T1), one case with size > 40 mm (T3), and one case with size > 40 mm invading adjacent structures (T4). Besides this, three cases showed the absence of regional lymph node metastasis (N0), one case showed metastasis in a single ipsilateral lymph node > 30 mm and ≤ 60 mm (N2), and one case showed metastasis in a single ipsilateral lymph node > 60 mm (N3). Regarding distant metastasis, four cases presented the absence (M0), and one showed the presence (M1) of distant metastasis. About the clinical stage, SCC frequently exhibited clinical stage I (39.8%; *n* = 62) and II (33.9%; *n* = 53) (Fig. 2), while BCC mainly presented stage IV (40%; *n* = 2) (Fig. 2). No significant association was observed between the anatomical location and the cancer clinical staging (*p* = 0.065) (Table 1).

Regarding the treatment approach of the neoplasms, SCC was mainly treated with surgery (88.3%; *n* = 264), followed by surgery with radiotherapy (11.4%, *n* = 34) and radiotherapy (0.3%; *n* = 1), while BCC was frequently treated with surgery (93.2%; *n* = 69), followed by surgery with radiotherapy (6.8%; *n* = 5). No significant association was observed between the anatomical location and the treatment (*p* = 0.674) (Table 1). The surgical margin was frequently negative in SCC and BCC (87%; *n* = 281; 72.3%; *n* = 68, respectively), and no recurrence was observed in 79.9% (n = 258) of SCC cases and 69.1% (n = 65) of BCC cases (Fig. 2). No significant association was observed between the type of neoplasm and the presence of regional lymph node metastasis, distant metastasis, treatment, cancer clinical stage, surgical margin, and recurrence.

## Discussion

Cancer is a public health problem worldwide, and its incidence has been increasing, with 354,864 new cases of oral cancer reported in 2018. Lip neoplasms differ etiologically from intraoral lesions since they are locations highly exposed to UV radiation (10-12). UVA and UVB rays act dose-dependently and cumulatively in initiating and promoting carcinogenesis due to direct damage to the DNA structure (13). The present study was conducted in two hospitals in northeast Brazil, a region close to the equator, making it more prone to UV radiation incidence and, consequently, contributing to increased skin cancer (13).

SCC and BCC of the lips are malignant neoplasms arising from keratinocytes, mainly from chronic UV radiation exposure. Besides, SCC mostly affects male patients between the fifth and sixth decades of life (5,14). In the present study, 48% of the patients reported an outdoor occupational activity related to chronic sun exposure, and 58.8% of patients diagnosed with SCC were males between the sixth and eighth decades of life. Our results are similar to other Brazilian studies, indicating that most patients are affected after the fourth decade of life (7,15,16).

Studies indicate that SCC frequently affects the lower lip, while BCC is rare in the lower lip and usually affects the upper lip (7,15,16-18). These results differ from the present study since we observed that SCC and BCC presented a significant preference for the lower lip, and the frequency of carcinomas in the lower lip was significantly higher in male patients. We believe this change in the epidemiological profile of lip BCC is due to the extension of the skin tumor to the lower lip, causing a greater prevalence in this anatomic site. As it is known, UVB is the main BCC predisposing factor, and it increases its development risk by 1.5-fold (17,18). In this way, this finding is explained by the anatomical lip position, which allows a higher incidence of UV radiation in this anatomic site, causing the initiation and promotion of lip carcinogenesis. Also, lower frequency in female patients can be explained by using lipstick, which acts as a protective factor. At the same time, males may present more prolonged outdoor occupational sun exposure and later retirement compared with women (5,7,19).

Initially, lip SCC presents clinically as a plaque/crusted lesion that may exhibit extensive ulcers, raised edges, and/or signs of infiltration in advanced stages. In contrast, head and neck BCC usually presents as a well-defined papule or nodule of slow growth with a pearly and telangiectatic central area, which may show ulcerated and pigmented areas (20,21). Our study observed that SCC was mainly clinically described as an ulcer or plaque and BCC as a nodule or papule. In this context, the differential diagnosis of SCC and BCC will vary according to the clinical presentation of the lesion since plaque, crusted, and ulcerated lesions perform differential diagnosis with herpes simplex at a later stage and traumatic ulcers. On the other hand, papular and nodular lesions may resemble trichoepithelioma, while pigmented lesions perform differential diagnosis with melanoma, seborrheic keratosis, and nevus (22,23).

Most cases of SCC and BCC of the lip are diagnosed in the initial clinical stages, exhibiting a small size, absence of metastasis in the lymph nodes, and distant metastasis (10,15). Most cases were classified as T1/T2 in the present study. This can be justified by the anatomical site affected by these carcinomas since the lip is an anatomical structure with aesthetic appeal, and the patient can quickly notice changes in it, leading the patient to seek clinical care and treatment (1,24). Furthermore, these neoplasms are characterized by less aggressive clinical behavior when they affect the lips (8,10), which may also explain the absence of a positive margin in most cases in the present study.

It is known that, in general, SCC exhibits more aggressive clinical behavior than BCC. In the initial stages, the treatment of these neoplasms consists of surgical resection; however, radio and chemotherapy may be necessary to treat advanced clinical stages of these lesions (1,7,8,20,25-27). Our results showed that surgery was the most frequent treatment approach for SCC and BCC. Lip SCC exhibits a 5-year survival rate of 82.1% and a distant metastasis rate of 2-10%, depending on cell differentiation, location, and tumor size. In contrast, the 5-year survival rate of lip BCC exceeds 90% due to the low rates of distant metastases (0.1%) (1,7,8,16,25-27). Our results show only 12.4% and 9.6% of recurrences in SCC and BCC cases, respectively. Although survival rates are favorable in this anatomical site, treatment-related consequences can compromise the patient’s quality of life once surgical treatment affects essential functions, such as chewing, swallowing, and phonation. Besides, it may cause aesthetic consequences, interfering with the lip-image self-perception of the patient and, consequently, the patient's self-esteem (1,26). Thus, it is crucial to perform prevention campaigns to raise awareness of using sunscreen and hats, especially in tropical countries, due to the high risk of chronic exposure to UV radiation (26).

Our study investigated the occurrence of lip cancer in two reference hospitals in diagnosing dermatological lesions and oncological treatment in northeastern Brazil. Although our results may be the basis for more studies that aim to evaluate the clinicopathological characteristics of lip cancer, some limitations of our study need to be considered. It is important to emphasize that the analyzed data from the present study represent a specific sample of a Brazilian location. Also, the lack of information, such as the TNM grading system of BCC cases, is a limitation. Despite this, we highlight that multicenter studies help the understanding of occurrence profiles of these neoplasms and can cover the heterogeneity of the population, especially in countries of continental dimensions such as Brazil.

In conclusion, our study showed that SCC was more frequent in male patients and BCC in female patients. Lesions in the lower lip were significantly frequent in male patients, and both SCC and BCC significantly affected this anatomical site. SCC was more frequent in patients between the sixth and eighth decades of life and BCC in the eighth decade of life. Besides, understanding the epidemiological profile and etiology of these lesions in the lip is essential for planning preventive measures in health education since the lip is an important functional and aesthetic region, possessing a strong relationship with the quality of life and individual self-esteem.

## Figures and Tables

**Table 1:** Association between clinicopathologic characteristics and anatomic site.

Clinicopathologic characteristics		Anatomic Site		p
		Lower lip n (%)	Upper lip n (%)	
Sex	Male	188 (82.1)	41 (17.9)	< 0.0001^a^*
	Female	93 (51.7)	87(48.3)	
Neoplasm	Squamous cell carcinoma	228 (71.7)	90 (28.3)	0.014^a^*
	Basal cell carcinoma	53 (58.2)	38 (41.8)	
Clinical Stage	Stage 0	3 (100)	0 (0)	0.065^a^
	Stage I	45 (71.4)	18 (28.6)	
	Stage II	44 (81.5)	10 (18.5)	
	Stage III	8 (53.3)	7 (46.7)	
	Stage IV	22 (88)	3 (12)	
Treatment	Surgery	225 (69)	101 (31)	0.674^a^
	Surgery + Radiotherapy	28 (73.7)	10 (26.3)	
	Radiotherapy	1(100)	0 (0)	

(a) Pearson's chi-squared test, (*) Result is statistically significant.
